# Long Sleep Duration Is an Independent Risk Factor for Incident Atrial Fibrillation in a Chinese Population: A Prospective Cohort Study

**DOI:** 10.1038/s41598-017-04034-8

**Published:** 2017-06-16

**Authors:** Qiaofeng Song, Xiaoxue Liu, Wanning Hu, Wenhua Zhou, Aijuan Liu, Xizhu Wang, Shouling Wu

**Affiliations:** 1 Department of Cardiology, Tangshan People’s Hospital, North China University of Science and Technology, Tangshan, China; 2 The Cancer Institute, Tangshan People’s Hospital, Tangshan, China; 3 Department of Medical, Tangshan People’s Hospital, North China University of Science and Technology, Tangshan, China; 4 Department of Cardiology, Kailuan Hospital, North China University of Science and Technology, Tangshan, China

## Abstract

There is limited information on the relation between sleep duration and incident atrial fibrillation. We aimed to investigate this association in a Chinese population using cohort data from a study in Kailuan. The analysis included 87,693 participants (age range, 18–98 years) free of atrial fibrillation at the baseline survey. Participants were divided into three categories according to self-reported sleep duration: ≤6.0 hours, 7 hours (ref), ≥8.0 hours. Atrial fibrillation diagnosis was made on a standard 12-lead electrocardiogram and via self-reported history. Cox proportional hazards models were used to calculate hazard ratio (HR) and confidence interval (CI) for atrial fibrillation, according to sleep duration. During median follow-up of 7.89 (range, 6.36–8.57) years, 322 cases of atrial fibrillation had occurred. Using 7 hours of sleep as the reference group, multivariable adjusted HRs (95% CI) for atrial fibrillation were 1.07 (0.75–1.53), 1.0 (ref), and 1.50 (1.07–2.10), from lowest to highest category of sleep duration. Secondary analysis showed no evidence of interactions between sleep duration and sex and snoring on the risk of incident atrial fibrillation (p = 0.75/0.25). We conclude long sleep duration may be a potential predictor/marker for incident atrial fibrillation.

## Introduction

Atrial fibrillation (AF) is the most common sustained arrhythmia, and is projected to affect 6–12 million people by 2050 in the United States and 17.9 million by 2060 in Europe^1–3^. In China, AF affects an estimated 3.9 million (2%) individuals aged ≥60 years^4^ and, by 2050, this is projected to rise to 9 million in this population, which will have reached 460 million^5^. AF represents a major public health problem, accompanying increased risks of stroke^6, 7^, myocardial infarction^8, 9^, heart failure^7, 10^, and chronic kidney disease^11, 12^, as well as increased mortality^7, 13^. Identifying all the risk factors for AF will help create population-based strategies for dealing with it.

Sleep duration is a risk factor for adverse health outcomes including hypertension^14, 15^, diabetes^16, 17^, obesity^18, 19^, dyslipidaemia^20^, metabolic syndrome^21, 22^, myocardial infarction^23, 24^, stroke^25, 26^, and total mortality^27, 28^. Little evidence on the association between sleep duration and incident AF has been found in research on a general population. The US-based Multi-Ethnic Study of Atherosclerosis found that greater slow-wave sleep time was significantly associated with lower odds of contracting AF^29^. Recent data from the US-based Physicians’ Health Study showed a modestly elevated risk of AF associated with long sleep duration^30^. Additionally, shorter sleep duration was associated with higher risk of AF in those with prevalent sleep apnoea. However, that study were only conducted in the United States, and generalizability to a Chinese population is uncertain. Therefore, the current study was designed to explore the association between sleep duration and AF in a general population in China. The study also analysed the effects of sex and of snoring on the independent association.

## Results

At baseline, participants were aged 18–98 (mean, 50.54) years. Those who reported sleeping for ≤6 h, 6–8 h, or ≥8 h per night were 24.16%, 19.43%, and 56.41%, respectively. Table 1 shows baseline characteristics according to sleep duration. Significant associations were found among sleep duration and age, sex, education level, smoking status, drinking status, physical activity, hypertension, diabetes mellitus, dyslipidaemia, myocardial infarction, and snoring status.

**Table 1:** Baseline characteristics according to sleep duration.

Variable	Sleep duration (hours)				p value
	Total (n = 87693)	≤6.0 (n = 21191)	7 (n = 17035)	≥8.0 (n = 49467)	
Age, years	50.54 ± 11.96	52.95 ± 11.55	50.44 ± 12.14	49.54 ± 11.92	<0.01
Gender male, n (%)	68974 (78.65)	17767 (83.37)	13582 (79.73)	37725 (76.26)	<0.01
High school or above, n (%)	18344 (20.92)	4741 (22.37)	5364 (31.49)	8239 (16.66)	<0.01
Current smoker, n (%)	30489 (34.77)	10487 (49.49)	8087 (47.47)	11915 (24.09)	<0.01
Current alcohol, n (%)	33375 (38.06)	11470 (54.13)	9091 (53.37)	12814 (25.90)	<0.01
Active physical activity, n (%)	13680 (15.60)	5133 (24.22)	4074 (23.92)	4473 (9.04)	<0.01
Snoring, n (%)	12455 (14.20)	4933 (23.28)	3353 (19.68)	4169 (8.43)	<0.01
Hypertension, n (%)	37594 (42.87)	9619 (45.39)	6750 (39.62)	21225 (42.91)	<0.01
Diabetes mellitus, n (%)	7777 (8.87)	2167 (10.23)	1482 (8.70)	4128 (8.34)	<0.01
Dyslipidemia, n (%)	31201 (35.58)	8376 (39.53)	6365 (37.36)	16460 (33.27)	<0.01
Myocardial infarction, n (%)	1051 (1.20)	463 (2.19)	236 (1.39)	352 (0.71)	<0.01
Body Mass Index, kg/m^2^	25.07 ± 3.47	25.09 ± 3.42	25.09 ± 3.43	25.05 ± 3.50	0.06
Systolic blood pressure, mmHg	130.24 ± 20.69	131.49 ± 20.67	129.27 ± 20.52	130.04 ± 20.73	<0.01
Diastolic blood pressure, mmHg	83.37 ± 11.69	83.55 ± 11.50	82.58 ± 11.37	83.57 ± 11.88	<0.01
Fasting blood glucose, mmol/L	5.46 ± 1.62	5.48 ± 1.66	5.42 ± 1.51	5.46 ± 1.64	0.43
Total cholesterol, mmol/L	4.96 ± 1.15	5.00 ± 1.20	4.97 ± 1.21	4.93 ± 1.11	<0.01
Triglycerides, mmol/L	1.68 ± 1.38	1.70 ± 1.39	1.67 ± 1.37	1.67 ± 1.38	0.04
Low-density lipoprotein, mmol/L	2.36 ± 0.88	2.46 ± 0.88	2.46 ± 0.88	2.29 ± 0.88	<0.01
High-density lipoprotein, mmol/L	1.54 ± 0.40	1.55 ± 0.40	1.52 ± 0.39	1.55 ± 0.39	<0.01
Uric acid, µmol/L	288.9 ± 83.0	299.8 ± 85.6	301.4 ± 87.0	280.0 ± 79.3	<0.01
High sensitivity C-reactive protein, mg/L	0.79 (0.30–2.0)	0.80 (0.30–1.94)	0.80 (0.30–1.90)	0.76 (0.28–2.09)	0.33

Compared with participants without AF, those with AF were significantly older, contained a higher percentage of men, had higher BMI, higher uric acid, higher sensitivity C-reactive protein, higher prevalence of hypertension, higher prevalence of diabetes mellitus or myocardial infarction, and had a more elevated snoring status. (Table 2).

**Table 2:** Differences in baseline characteristics between patients with and without atrial fibrillation.

Variable	Atrial Fibrillation	No Fibrillation	p value
Age, years	61.57 ± 10.20	50.50 ± 11.94	<0.01
Gender male, n (%)	268 (83.23)	68706 (78.64)	0.04
High school or above, n (%)	47 (14.60)	18297 (20.94)	<0.01
Current smoker, n (%)	88 (27.33)	30401 (34.80)	<0.01
Current alcohol, n (%)	107 (33.23)	33268 (38.08)	0.07
Active physical activity, n (%)	75 (23.29)	13605 (15.57)	<0.01
Snoring, n (%)	63 (19.57)	12392 (14.18)	<0.01
Hypertension, n (%)	219 (68.01)	37375 (42.78)	<0.01
Diabetes mellitus, n (%)	39 (12.11)	7738 (8.86)	0.04
Dyslipidemia, n (%)	120 (37.27)	31081 (35.57)	0.53
Myocardial infarction, n (%)	13 (4.05)	1038 (1.19)	<0.01
Body Mass Index, kg/m^2^	26.15 ± 3.89	25.06 ± 3.47	<0.01
Systolic blood pressure, mmHg	141.08 ± 21.24	130.20 ± 20.68	<0.01
Diastolic blood pressure, mmHg	86.36 ± 11.31	83.36 ± 11.69	<0.01
Fasting blood glucose, mmol/L	5.46 ± 1.65	5.46 ± 1.62	0.28
Total cholesterol, mmol/L	4.89 ± 1.08	4.96 ± 1.15	0.22
Triglycerides, mmol/L	1.72 ± 1.19	1.68 ± 1.38	0.05
Low-density lipoprotein, mmol/L	2.18 ± 0.86	2.36 ± 0.88	<0.01
High-density lipoprotein, mmol/L	1.59 ± 0.44	1.54 ± 0.40	0.18
Uric acid, µmol/L	313.82 ± 86.64	288.85 ± 83.02	<0.01
High sensitivity C-reactive protein, mg/L	1.18 (0.46–3.20)	0.78 (0.30–2.00)	<0.01

Table 3 shows the hazard ratios (HRs) for AF according to sleep duration in the total population and stratified by sex. A total of 322 cases of AF had occurred as of the median follow-up (7.89 years): 268 in men, and 54 in women. Crude incidence rates of AF were 0.54, 0.42, and 0.53 cases/1,000 person-years for people reporting average sleep duration of ≤6, 7, and ≥8 hours, respectively. Using 7 hours of sleep as the reference group, multivariable adjusted HRs (95% CI) for AF were 1.07 (0.75–1.53), 1.0 (ref), and 1.50 (1.07–2.10) from lowest to highest category of sleep duration, respectively (Table 3). Men who slept ≥8 hours were in fact found more likely to develop AF (HR, 1.46; 95% CI, 1.02–2.10). While this association was not significant among women (HR, 1.69; 95% CI, 0.70–4.11), a formal test for difference by sex also did not find statistical significance (p = 0.75).

**Table 3:** Hazard ratios (95% CI) for atrial fibrillation according to sleep duration in the Kailuan Study.

Total	Sleep duration (hours)			P for interaction
	≤6.0	7	≥8.0	
Case, incidence, per 1000 person-years	82 (0.54)	51 (0.42)	189 (0.53)	
Model 1	1.10 (0.78–1.57)	reference	1.47 (1.08–2.00)	0.67
Model 2	1.09 (0.77–1.55)	reference	1.47 (1.06–2.05)	0.68
Model 3	1.07 (0.75–1.53)	reference	1.50 (1.07–2.10)	0.75
**Women**				
Case, incidence, per 1000 person-years	15 (0.57)	7 (0.27)	32 (0.37)	
Model 1	1.47 (0.60–3.60)	reference	2.12 (0.93–4.83)	
Model 2	1.43 (0.58–3.54)	reference	1.71 (0.71–4.12)	
Model 3	1.28 (0.51–3.20)	reference	1.69 (0.70–4.11)	
**Men**				
Case, incidence, per 1000 person-years	67 (0.54)	44 (0.45)	157 (0.59)	
Model 1	1.04 (0.71–1.52)	reference	1.38 (1.00–1.93)	
Model 2	1.03 (0.70–1.51)	reference	1.43 (1.01–2.05)	
Model 3	1.03 (0.70–1.51)	reference	1.46 (1.02–2.10)	

Model 1: Adjusted for age and sex. Model 2: Adjusted for age, sex, level of education, smoking, alcohol, physical activity, snoring, and body mass index. Model 3: Adjusted for variables in Model 2 plus hypertension, diabetes mellitus, dyslipidaemia, myocardial infarction, uric acid, and high-sensitivity C-reactive protein.

We further repeated the analysis stratified by different age groups (Table 4). Participants aged <60 years and who slept ≥8 hours were found likely to develop AF (HR, 1.86; 95% CI, 1.09–3.18). While this association was not significant among participants ≥60 years (HR, 1.30; 95% CI, 0.84–2.00), another test for difference by sex also did not find statistical significance (p = 0.14).

**Table 4:** Hazard ratios (95% CI) for atrial fibrillation according to sleep duration stratified by age in the Kailuan Study.

	Sleep duration (hours)			P for interaction
	≤6.0	7	≥8.0	
**Age < 60**				
Case, incidence, per 1000 person-years	23 (0.20)	18 (0.18)	96 (0.32)	
Model 1	0.99 (0.54–1.84)	reference	1.95 (1.18–3.22)	0.10
Model 2	1.00 (0.54–1.85)	reference	1.87 (1.10–3.19)	0.09
Model 3	0.95 (0.51–1.78)	reference	1.86 (1.09–3.18)	0.14
**Age >= 60**				
Case, incidence, per 1000 person-years	59 (1.67)	33 (1.44)	93 (1.71)	
Model 1	1.13 (0.74–1.73)	reference	1.22 (0.82–1.82)	
Model 2	1.10 (0.72–1.69)	reference	1.25 (0.81–1.92)	
Model 3	1.10 (0.71–1.70)	reference	1.30 (0.84–2.00)	

Model 1: Adjusted for age and sex. Model 2: Adjusted for age, sex, level of education, smoking, alcohol, physical activity, and body mass index. Model 3: Adjusted for variables in Model 2 plus hypertension, diabetes mellitus, dyslipidaemia, myocardial infarction, uric acid, and high-sensitivity C-reactive protein.

In a secondary analysis, sleeping ≥8 hours combined with snoring showed a significantly increased risk of AF (HR, 2.05; 95% CI, 1.04–4.04). There was no evidence for an interaction between sleep duration and snoring on the risk of incident AF (p = 0.25, Table 5).

**Table 5:** Hazard ratios (95% CI) for atrial fibrillation according to sleep duration stratified by snoring status in the Kailuan Study.

Snoring	Sleep duration (hours)			P for interaction
	≤6.0	7	≥8.0	
Case, incidence, per 1000 person-years	22 (0.63)	12 (0.50)	29 (0.98)	
Model 1	1.12 (0.55–2.26)	reference	2.11 (1.08–4.13)	0.41
Model 2	1.07 (0.52–2.17)	reference	2.06 (1.04–4.06)	0.36
Model 3	1.03 (0.45–1.93)	reference	2.05 (1.04–4.04)	0.25
**NO Snoring**				
Case, incidence, per 1000 person-years	60 (0.51)	39 (0.40)	160 (0.49)	
Model 1	1.10 (0.73–1.64)	reference	1.39 (0.98–1.97)	
Model 2	1.09 (0.73–1.64)	reference	1.30 (0.90–1.89)	
Model 3	1.12 (0.74–1.68)	reference	1.35 (0.93–1.97)	

Model 1: Adjusted for age and sex. Model 2: Adjusted for age, sex, level of education, smoking, alcohol, physical activity, and body mass index. Model 3: Adjusted for variables in Model 2 plus hypertension, diabetes mellitus, dyslipidaemia, myocardial infarction, uric acid and high-sensitivity C-reactive protein.

## Discussion

In this prospective, population-based, cohort study, long sleep duration independently predicted increased risk for incident AF as shown during a median 7.89 years of follow-up. In secondary analysis, the prolong sleep duration was significantly associated with a higher risk of AF in people who snored, but not in non-snorers. This relationship persists independently of other known major risk factors such as smoking, alcohol, diabetes, hypertension, dyslipidaemia, obesity, myocardial infarction, uric acid, and high-sensitivity C-reactive protein.

The US-based Physicians’ Health Study^30^, conducted on 18,755 male physicians, showed that long sleep duration increased risk of AF by 13% compared with participants who slept 7 hours. Our results also demonstrated long sleep duration was an independent risk factor for incident AF. The increased risk for AF in our study was a much higher figure of 50%. Additionally, sex differences in classical AF risk factors known from prior reports persisted during the observational period^31^. We therefore also tested, using interaction regression models, the difference in the association of sleep duration with AF between men and women. The significant association between long sleep duration and AF was found in men, but not in women, while there was no evidence of effect modification by sex (p interaction = 0.751). This inconsistency may be due to low AF incidence and the small sample size of women. Indeed, the positive association between sleep duration and AF may also exist in women (though it may not be statistically significant). Considering that sleep behaviours differing between younger and older participants could have biased the association, we further repeated the analysis, stratified by age group. However, no evidence was found of interaction between sleep duration and age on the risk of incident AF (p interaction = 0.14).

Previous studies demonstrated sleep deprivation could be an important AF predictor as assessed by electrocardiogram parameters^32, 33^. The Physicians’ Health Study^30^ also found short, but not long, sleep duration was associated with a higher risk of AF in people with sleep apnoea. In contrast, in our secondary analysis, snoring status was used for the secondary analysis instead of sleep apnoea. We observed a significantly higher risk of AF among snorers and long sleep duration. Among individuals with short sleep duration and snoring, there was an evident trend towards increased AF risk (not statistically significant). These inconsistent results may be due to the different populations in the studies. Additionally, use of snoring status in our study was for secondary analysis rather than sleep apnoea, which was used in the Physicians’ Health Study, may have lead to different results.

The real pathophysiologic mechanisms that mediated the link between long sleep duration and AF are not known and require further exploration. There are several potential explanations for why prolong sleep duration may be a risk factor for incident AF. Previous studies indicate that longer sleep duration may be reflective of low socioeconomic status, depression, low level of physical activity, and periods of failing health and illness^34, 35^. Sleep also reflects high parasympathetic and low sympathetic activity^36^, and we can postulate that people with long sleep duration have prolonged exposure to increased vagal tone (parasympathetic), which has been found associated with induction and maintenance of AF^37^.

Our study has several strengths as is the first prospective study to address the association between sleep duration and incidence AF in a general population in China. However, there are some inherent limitations. First, sleep duration data were collected via self-reported questionnaires; data on midday naps and sleep quality were not undertaken in our study. We also did not exclude participants with sleep apnoea, which is associated with high rates of AF^38^. However, we adjusted snoring status as a confounder in the statistical analysis. Second, AF in our study was diagnosed based on a single electrocardiogram, without ambulatory electrocardiogram monitoring. Combined with AF can be permanent, but in the beginning is often paroxysmal. Furthermore, no asymptomatic patients were seen at the hospital. We acknowledge that the incidence of AF was undoubtedly underestimated. This diagnostic approach to AF has been used in previous studies^30, 39^. Third, we only investigated the association between sleep duration at baseline examination and future AF, without taking change in sleep duration into consideration. Indeed, any subsequent change in sleep duration could lead to non-differential misclassification and potentially underestimate the sleep–AF association. Finally, all participants were employees of the Kailuan Coal Company, and most were men; therefore, they cannot be viewed as a representative sample of the general Chinese population. But they have a complicated constitution from all levels of the society whose occupation may be coalminers, administrators, secretaries, accountants, as well as the supportive and service staff, such as policemen, doctors, nurses, vendors, teachers, etc. And studying such a geographically focused and controlled population greatly reduces residual confounding owing to diverse socioeconomic factors and lifestyle patterns.

In conclusion, our findings from the Kailuan cohort study suggest that long sleep duration may cause increased risk of AF in a Chinese population. Encouraging and supporting individuals to pursue 7 hours of sleep per night may have significant beneficial effects towards stemming the growing prevalence of AF in China.

## Methods

### Ethics Statement

The protocol for the study was approved by the Ethics Committee of Kailuan General Hospital in compliance with the Declaration of Helsinki. All participants provided informed written consent with their signatures.

### Study design and participants

The Kailuan study was a prospective cohort study conducted in the Kailuan community in Tangshan City, China. It is a large comprehensive community in Tangshan city (the city of Tangshan is situated 150 km southeast of Beijing and represents the Chinese population from a socioeconomic perspective) of more than 120 years of history that have a low population mobility and relative internal stability, owning schools, hospitals, police stations, shopping centers, hotels, and so on^40–42^. From June 2006 to October 2007, 101,510 (81,110 men, 20,400 women; age range, 18–98 years) employees (including retired persons) in the community agreed to enrol in study. Participants underwent health examinations biennially until December 31, 2015. Individuals who participated in at least one follow-up examination in the 2008–2009, 2010–2011, 2012–2013, and 2014–2015 examination circles (n = 91,542) were included. Additionally, 313 individuals with diagnosed pre-existing AF, and 3,536 individuals with incomplete sleep duration data, were excluded. The remaining 87,693 participants free of AF were included in the final analysis.

### Assessment of sleep duration

Sleep duration data were obtained through self-reported responses to the question, “How many hours of sleep have you had on average night in the preceding 3 months?” Possible responses were “≤5 hours”, “6 hours”, “7 hours”, “8 hours”, and “≥9 hours”. Based on the responses, sleep durations were categorized into three groups: short (≤6 hours), average (7 hours), and long (≥8 hours). Participants were also asked the yes-or-no question “Do you generally snore when you sleep?”

### Follow-up and AF assessment

Participants were followed up via face-to-face interviews at every 2-year routine medical examination until December 31, 2015, or until the event of interest or death. Diagnosis of AF was made using a standard 12-lead electrocardiogram^39^. Participants were also diagnosed with AF if atrial flutter was present on the electrocardiogram readings.

### Assessment of potential covariates

Demographic and clinical characteristics, including age, sex, alcohol use, education, and disease history, were collected via self-reported questionnaires. Educational attainment was categorized as “illiterate or primary school”, “middle school”, or “high school or above”. Information on physical activity level (minutes of moderate or vigorous activity per week) was obtained from questionnaires and categorized as follows: ≥80 (ideal); 1 to 79 (intermediate) and; 0 (poor) minutes of moderate or vigorous activity per week^42^. Smoking status and drinking status were classified as “never”, “former”, or “current” according to self-reported information. Body mass index was calculated as kg/m^2^. Systolic blood pressure and diastolic blood pressure were measured three times in a seated position and using a mercury sphygmomanometer. All blood samples were tested using a Hitachi 747 auto-analyser (Hitachi; Tokyo, Japan) at the central laboratory of the Kailuan General Hospital. Fasting blood glucose, triglyceride, total cholesterol, high-density lipoprotein, low-density lipoprotein, serum uric acid, and high-sensitivity C-reactive protein levels were measured.

### Statistical Analyses

Continuous variables were described as mean ± standard deviation or median (interquartile range) and were compared using analysis of variance or the Kruskal–Wallis test. Categorical variables were described as percentages and were compared using chi-square tests. Person-years were calculated from the date the 2006 interview was conducted to the date when the first occurrence of AF was detected, date of death, or date of last attended interview in this analysis, whichever came first.

Cox proportional hazards regression was used to estimate the risk of AF by calculating the HR and 95% confidence interval, and using 7 hours of sleep duration as the reference group. Variables that were clinically significant or associated with outcomes in univariate analysis with p < 0.20 were included in the multivariable model. Model 1 adjusted for age and sex. Model 2 further adjusted for education level, drinking, smoking, physical activity, snoring, and body mass index. Model 3 further adjusted for history of hypertension, diabetes mellitus, dyslipidaemia, myocardial infarction, uric acid, and high-sensitivity C-reactive protein. In secondary analyses, we evaluated whether there were statistically significant interactions between sleep duration and age/sex or snoring status. All interactions were analysed using multivariable Cox proportional hazards modelling. Statistical analyses were performed using SAS 9.4 (SAS Institute; Cary, NC). All statistical tests were two-sided, and the significance level was set at 0.05.
